# The diatom-derived aldehyde decadienal affects life cycle transition in the ascidian *Ciona intestinalis* through nitric oxide/ERK signalling

**DOI:** 10.1098/rsob.140182

**Published:** 2015-03-18

**Authors:** Immacolata Castellano, Elena Ercolesi, Giovanna Romano, Adrianna Ianora, Anna Palumbo

**Affiliations:** Stazione Zoologica Anton Dohrn, Villa Comunale, 80121 Naples, Italy

**Keywords:** decadienal, *Ciona*, metamorphosis, nitric oxide, mkp1, ERK

## Abstract

Polyunsaturated aldehydes (PUAs) are fatty-acid-derived metabolites produced by some microalgae, including different diatom species. PUAs are mainly produced as a wound-activated defence mechanism against microalgal predators or released from senescent cells at the end of a bloom. PUAs, including 2,4-*trans*-decadienal (DD), induce deleterious effects on embryonic and larval development of several planktonic and benthic organisms. Here, we report on the effects of DD on larval development and metamorphosis of the ascidian *Ciona intestinalis. Ciona* larval development is regulated by the cross-talking of different molecular events, including nitric oxide (NO) production, ERK activation and caspase 3-dependent apoptosis. We report that treatment with DD at the competence larval stage results in a delay in metamorphosis. DD affects redox balance by reducing total glutathione and NO levels. By biochemical and quantitative gene expression analysis, we identify the NO-signalling network affected by DD, including the upregulation of ERK phosphatase *mkp1* and consequent reduction of ERK phosphorylation, with final changes in the expression of downstream ERK target genes. Overall, these results give new insights into the molecular pathways induced in marine organisms after exposure to PUAs during larval development, demonstrating that this aldehyde affects key checkpoints of larval transition from the vegetative to the reproductive life stage.

## 2. Introduction

Diatoms are photosynthetic unicellular microalgae contributing up to 45% of total oceanic primary production and playing key roles in the transfer of energy through marine food chains [1]. The reason for their ecological success still remains a matter of discussion, and different mechanisms have been proposed to explain their widespread occurrence in the oceans. One main reason may be because they have evolved a chemical defence system which can potentially affect both predators and competitors. Indeed, different diatom species are able to produce a wide range of secondary metabolites belonging to the oxylipin family including polyunsaturated aldehydes (PUAs) with toxic effects on reproductive processes in grazing organisms such as crustacean copepods [2–4], echinoderms [5,6] and filter-feeding organisms such as ascidians [7]. These metabolites are the end-products of a lipoxygenase/hydroperoxide lyase metabolic pathway, initiated by damage of algal cells as would occur through grazing [8]. Briefly, cell damage activates lipase enzymes, causing the release of polyunsaturated fatty acids from cell membranes, which are then immediately oxidized and cleaved to form PUAs, including 2,4-*trans*-decadienal (DD). This aldehyde, in particular, is produced by some diatoms (e.g. *Thalassiosira rotula, Skeletonema subsalsum* and *Chaetoceros compressus*) [9] and by other microalgal groups such *as* Prymnesiophytes (*Phaeocystis pouchetti*) [10] and freshwater crysophytes [11]. PUAs have important biological and biochemical effects on marine organisms. Interestingly, they do not appear to be directly toxic to adults, but specifically affect gametogenesis, gamete functionality, fertilization, embryonic mitosis and larval stages [6,12,13]. Moreover, at high concentrations, such toxins can affect offspring survival, by inducing abortions, birth defects and reduced larval fitness and competence [2,3,14–16]. In this context, PUAs production has been proposed as a defence mechanism to negatively impact future generations of grazers, thereby allowing diatom blooms to persist when grazing pressure would otherwise have caused them to crash. However, PUAs can also be released at the end of a bloom, possibly acting as signalling molecules to terminate the bloom [10,17,18]. Moreover, physiological stress linked with nutrient availability has been recently proposed to modulate PUAs production also in natural non-blooming areas [19]. In the proximity of diatoms blooms, the released PUAs can create microzones where they can react with neighbouring organisms.

In the sea urchin *Paracentrotus lividus*, exposure to low concentrations of DD has been shown to induce teratogenesis and abnormal plutei formation during development, whereas high concentrations of DD block cell cleavage and induce apoptosis [20]. These effects are mediated by the physiological messenger nitric oxide (NO) [21].

The aim of this study was to investigate the effect of DD on larval development in the ascidian *Ciona intestinalis*, an excellent marine model system to study molecular pathways responsible for life cycle transitions because its genome is completely available together with information on the expression profiles of several developmental genes [22–24].This ascidian has a biphasic life cycle with a simple swimming tadpole larva and a sessile juvenile/adult. *Ciona* is a hermaphroditic broadcast spawner. When eggs and sperm are released, they can stay in the water column for 1–2 days, until fertilization occurs. After hatching, approximately 18 h post-fertilization (hpf), larvae swim for a few hours and develop into three larval stages: early, middle and late. At the middle-late stage, larvae become competent to sense environmental stimuli, and the complete formation of adhesive papillae allows them to attach to a suitable substrate, inducing metamorphosis (approx. 28 hpf) through a profound reconstruction of the body plan and a remarkable regression of the tail [25]. *C. intestinalis* larval development and initial stages of metamorphosis are regulated by different molecular events, sometimes cross-talking together. These events include: NO production/diffusion, activation of the extracellular-signal-regulated kinase (ERK) and the c-Jun NH_2_-terminal kinase (JNK), and caspase 3-mediated apoptosis [26–28]. Increases in NO and reactive nitrogen species levels accelerate metamorphosis, inducing a nitro-oxidative stress pathway resulting in ERK nitration [29]. Recently, we have shown that the NO-mediated acceleration of *Ciona* metamorphosis occurs through the downregulation of MAPK phosphatases mkp1 and mkp3 resulting in ERK activation [30]. The first peak of ERK activation during larval development occurs in papillae (20–22 hpf) and this, in turn, activates a series of downstream genes, probably involved in competence acquisition and the attachment of larvae to the substrate [28]. The second peak of ERK activation occurs at the onset of metamorphosis (28 hpf) and precedes the apoptotic wave necessary for the initiation of tail regression [27].

Here, we show that treatment of competent larvae with DD causes a reduction in glutathione (GSH) and NO levels. The decrease of endogenous NO is responsible for the upregulation of ERK phosphatase mkp1 with consequent dephosphorylation of ERK and changes in downstream ERK target gene transcription leading to delayed metamorphosis.

## 3. Material and methods

### 3.1. Chemicals

All reagents were purchased from Sigma-Aldrich, Milan, Italy, unless otherwise stated.

### 3.2. *Ciona intestinalis* incubation experiments

Adult *C. intestinalis* were collected at Fusaro Lake in the district of Naples (40°49′10.6″ north latitude, 14°0.3′ 32″ east longitude). No specific permissions were required for this location that is not privately owned or protected in any way. The study did not involve endangered or protected species, and was carried out in strict accordance with European (Directive 2010/63) and Italian (Decreto Legislativo no. 116/1992) legislation for the care and use of animals for scientific purposes. Animals were transported to the laboratory and maintained at 18°C in tanks with circulating seawater and under constant light to allow gamete accumulation. Animal handling and fertilization were carried out as previously described [29,30]. In brief, eggs from single animals were fertilized with a mixture of sperm obtained from different individuals. Embryos were cultured at 18°C in 0.2 µm filtered seawater. Hatched larvae were obtained at 18–20 hpf at 18°C. Development was followed on live specimens with a Stemi 2000 (ZEISS) stereomicroscope. Samples at appropriate stages were identified using the morphological criteria previously reported by Chiba *et al*. [31] and were selected on the basis of at least 95% homogeneity. Larvae (about 100 larvae ml^−1^) were treated in tissue culture dishes in 50 ml of seawater at 18°C with DD at final concentrations ranging from 0.125 (0.8 µM) to 1.35 µg ml^−1^ (8.9 µM) or with MKP inhibitor dusp1/6 I (Calbiochem, Merck Millipore, Milan, Italy) at 0.25 µM. Treated larvae were allowed to develop to the desired stage and were collected by low-speed centrifugation (500*g*) after washing in PBS buffer. Pellets were stored at −80°C until use and subsequently used for protein extraction, NO detection and RNA preparation.

### 3.3. Protein extraction

Larval pellets were homogenized in two volumes of RIPA lysis buffer (150 mM NaCl, 50 mM Tris–HCl pH 7.6, 5 mM EDTA, 0.5% NP-40, 0.5% sodium deoxycholate, 0.1% SDS) supplemented with protease inhibitors (1 mM PMSF and Complete Protease Inhibitor Cocktail Tablets, Roche, Monza, Italy) and phosphatase inhibitors (PhosSTOP Cocktail Tablets, Roche). After centrifugation, protein concentration was determined using a Bio-Rad protein assay reagent (Bio-Rad, Milan, Italy) with bovine serum albumin as a standard.

### 3.4. Fluorimetric determination of nitric oxide concentration

Larval pellets (50–60 mg) were homogenized in 0.5 ml of PBS and sonicated three times for 1 min at 30% amplitude. The samples were centrifuged at 13 500*g* for 5 min at 4°C, and the supernatants were collected for NO analysis using 2,3-diaminonaphthalene (DAN) to form the fluorescent product 1-(*H*)-naphthotriazole. Briefly, 80 µl of samples were incubated at 26°C with 0.06 U ml^−1^ nitrate reductase, 2.5 µM FAD and 100 µM NADPH for 1 h. Then, 10 μl of DAN (0.05 mg ml^−1^ in 0.62 M HCl) was added, and the sample was incubated for 20 min in the dark. After stabilization with 1N sodium hydroxide, formation of 1-(H)-naphthotriazole was measured using a spectrofluorimeter (Shimadzu RF-5301 PC) with excitation and emission at 365 and 425 nm, respectively. Calibration curves were measured daily with sodium nitrite (1–10 µM) in PBS. The experiments were carried out on three biological replicates.

### 3.5. Glutathione assay

Total GSH was determined by using the GSH assay kit (Sigma). Briefly, larval pellets (50 mg) were homogenized in 1 volume of PBS. An aliquot was used for protein determination, and another aliquot was added to 3 volumes of 5% 5-sulfosalicylic acid and mixed. Samples were then frozen (−80°C) and thawed at 37°C twice, left for 5 min at 4°C and finally centrifuged at 10 000*g* for 10 min. The supernatant was used for glutathione determination by performing a kinetic assay in which catalytic amounts (nmoles) of GSH caused a continuous reduction of 5,5′-dithiobis(2-nitrobenzoic acid) to 5-thio-2-nitrobenzoic acid measured spectrophotometrically at 405 nm with a Thermo Scientific™ Multiskan™ FC Microplate Photometer.

### 3.6. Immunoblot analysis

Larval lysates were prepared as previously described [29] and examined using 10% SDS–PAGE. The gel transferred to the nitrocellulose membrane was analysed with antibodies against p44/42 MAP kinase (ERK 1/2; ERK; 1 : 1000; Cell Signaling, EuroClone, Milan, Italy) and Phospho-p44/42 MAPK (P-ERK; 1 : 500; Cell Signaling). After washing in PBS with 0.1% Tween, labelled proteins were detected by ECL PLUS (GE Healthcare, EuroClone, Milan, Italy).

### 3.7. Bioinformatic analysis

*Ciona* transcripts were retrieved from Aniseed (http://www.aniseed.cnrs.fr/). The identification of transcripts was performed using the TBLASTX program.

### 3.8. RNA extraction and cDNA synthesis

Total RNA was extracted at different developmental stages using TRIZOL (Life Technologies, Milan, Italy) according to the manufacturer's instructions. Briefly, extraction with chloroform/isoamyl alcohol (24 : 1) was performed following RNA precipitation by addition of glycogen and isopropyl alcohol. Contaminating DNA was degraded by treating each sample with DNase (Roche) and then removing the enzyme with RNeasy MinElute™ Cleanup Kit (Qiagen, Milan, Italy). The amount of total RNA was estimated by the absorbance at 260 nm and the purity by 260/280 and 260/230 nm ratios, by Nanodrop (ND-1000 UV–Vis spectrophotometer; NanoDrop Technologies). The integrity of RNA was checked in agarose gel electrophoresis by visualizing intact rRNA subunits (28S and 18S). For each sample, 1 µg of total RNA extracted was retrotranscribed with iScript™ cDNA synthesis kit (Bio-Rad), following the manufacturer's instructions. cDNA was diluted 1 : 10 with H_2_O prior to use in real-time qPCR experiments.

### 3.9. Gene expression by real-time qPCR

For real-time qPCR experiments, the data from each cDNA sample were normalized using ribosomal protein R27a as a reference gene, the levels of which remained relatively constant in all the developmental stages examined and during treatment with DD. For each target gene, specific primers were designed on the basis of nucleotide sequences with the help of Primer3web v. 4.0.0 (table 1). For some genes, we used previously reported primers [28]. The amplified fragments using Taq high fidelity PCR system (Roche) were purified from agarose gel using QIAquick gel extraction kit (Qiagen) and specificity of PCR products was checked by DNA sequencing (Molecular Biology Service, SZN, Naples). Specificity of amplification reactions was verified by melting curve analysis. The efficiency of each primer pair was calculated according to standard method curves using the equation *E* = 10^−1/slope^. Five serial dilutions were set up to determine *C*_t_ values and reaction efficiencies for all primer pairs. Standard curves were generated for each oligonucleotide pair using the *C*_t_ values versus the logarithm of each dilution factor. PCR efficiencies were calculated for control and target genes and were found to be 2 in most cases, with the exception of *gclm* and *glrx* with values of 1.92 and 1.97, respectively. Diluted cDNA was used as a template in a reaction containing a final concentration of 0.3 µM for each primer and 16 FastStart SYBR Green master mix (total volume of 10 µl). PCR amplifications were performed in a ViiATM 7 real-time PCR system (Applied Biosystems) thermal cycler using the following thermal profile: 95°C for 10 min, one cycle for cDNA denaturation; 95°C for 15 s and 60°C for 1 min, 40 cycles for amplification; 72°C for 5 min, one cycle for final elongation; one cycle for melting curve analysis (from 60°C to 95°C) to verify the presence of a single product. Each assay included a no-template control for each primer pair. To capture intra-assay variability, all real-time qPCR reactions were carried out in triplicate. Fluorescence was measured using the ViiATM 7 software (Applied Biosystems). The expression of each gene was analysed and internally normalized against R27a using relative expression software tool (REST) software based on the Pfaffl method [34,35]. Relative expression ratios above twofold were considered significant. Experiments were repeated at least three times.

**Table 1.** Features of transcripts used for gene expression analysis: gene acronyms, accession numbers, primer sequences and information on gene regulation in *Ciona*.

gene acronym	accession number	gene name	gene regulation	primer sequences (5′–3′)
*R27a*	KYOTOGRAIL2005.209.6.1	ribosomal protein	reference	For**-**ATCCACCCTTCACCTTGTG
				Rev-GGAGATCTTGCCATTTTCA
*dhg*	ci0100130315	dehydrogenase with different specificity	ERK- regulated [28]	For-GTCACCGTTTCCTCTGAAGC
				Rev-GCGCCGTGTATTATGGTCTT
*ets*	ci0100152617	Ets-related transcription factor	ERK- regulated [28]	For-GACGAAGCATGTGTACCTGAC
				Rev-GCGGTTTTTCTGCACACCCC
*gclm*	XM_002128788.2	glutamate–cysteine ligase regulatory subunit	stress-regulated [32]	For-AGCCAATCTGGAAGGAGATGCAGT
				Rev-TTCTGCAGGGTCGTTATGGGTCAA
*glrx*	ENSCING00000014681 [33]	glutaredoxin		For-GCTCTACCCAGCTATGCCAGAA
				Rev-ACCACCTCCAATGCTTTGACC
*ggt*	ci0100152395	gamma-glutamyl transpeptidase		For-GCCAAGGAAGGAGCGTCTGT
				Rev-GGGGTCCTGGTGAAAACACG
*gss*	ENSCING00000009385 [33]	glutathione synthase	stress-regulated [32]	For-TCGCCACTTTCCAACACCA
				Rev-TGTTTCCTCCACCTTCTCG
*gstm*	ENSCING00000015785 [33]	glutathione transferase		For-AGAGGGTTAGGCGAACCTATTCGT
				Rev-CCAAACTCCGTTGCAGTTGTTCCA
*mkp1*	ci0100138796	dual-specific MAPK phosphatase 1	NO-regulated [30]	For-CCACTTTCCAGACCGATTTC
				Rev-CCTCACAGGTCCACTCCATT
*mkp3*	ci0100140262	dual-specific MAPK phosphatase 3	NO-regulated [30]	For-CGATGTTGGCGTTGCTGTAC
				Rev-GATGGACGGAGCATGAATGG
*MnSOD*	ci0100142427	manganese superoxide dismutase		For-GGTGGATCTGAGCCTTGTGGA
				Rev-GGGCAAGCCACGACCTGTAA
*mx*	ci0100149359	matrix metalloprotease	ERK-regulated [28]	For-GATTCCCAGCTAGTATCCG
				Rev-CGTTCTTCTGCTTGGATTGT
*NOS*	ci0100153759	nitric oxide synthase		For-AGAGTGAAAGCCTGTCGCATA
				Rev-AACCAATGCGGTGGTTGTAG
*sushi*	ci0100139289	cell adhesion molecule	JNK-regulated [28]	For-CAAGCATGCGAAGCGAATGAGGCTC
				Rev- GCAGCAACAACGACATACAGAG

### 3.10. Statistical analysis

Statistical analysis was performed using GraphPad Prism v. 4.00 for Windows (GraphPad Software, San Diego, CA). The results were reported as means ± s.e.m. and analysed by unpaired *t*-test for comparison between the groups; *p* < 0.05 was considered statistically significant. For real-time qPCR analysis, significance was tested using the ‘pairwise fixed reallocation randomization test’, developed by REST software [35]. The number of experiments is reported in the figure legends.

## 4. Results

### 4.1. Decadienal affects metamorphosis of *Ciona intestinalis*

**Figure 1.**
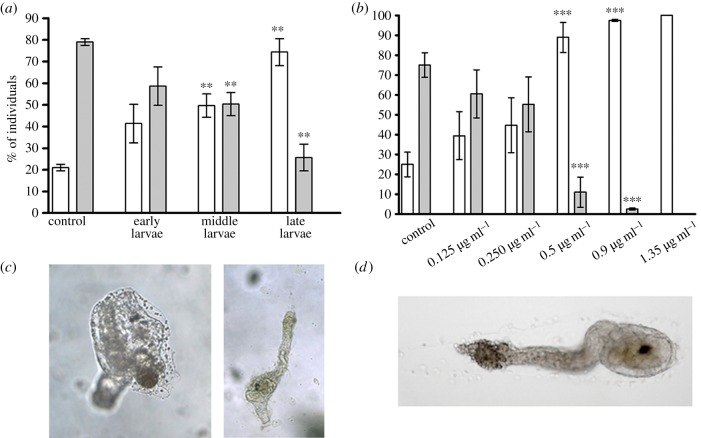
Decadienal (DD) induces a delay in metamorphosis at the time of competence. (*a*) Early, middle and late stage larvae were treated with 0.5 µg ml^−1^ DD. (*b*) Middle-late stage larvae were treated with different concentrations of DD (0.125, 0.25, 0.5, 0.9, 1.35 µg ml^−1^). After 24 h of treatment, the number of late larvae, larvae during tail regression (white bars) and juveniles (grey bars) were counted and reported as percentage of the total. Results are representative of 7 (*a*) and 10 (*b*) independent experiments. Data, expressed as means ± s.e.m., were assessed by unpaired *t*-test. Asterisks represent significant differences with respect to the control: **p < 0.01, ***p < 0.001. (*c*) Left panel shows untreated control juvenile and right panel shows abnormal juvenile after 72 h of incubation with 0.5 µg ml^−1^ DD. (*d*) Larva treated with 1.35 µg ml^−1^ DD after 24 h of treatment (toxic effect).

To investigate the effect of DD on *C. intestinalis* larval settlement and subsequent metamorphosis, larvae were treated at different times from hatching, corresponding to early, middle and late larval stages. Larvae were morphologically analysed after 24 h treatment. DD did not significantly affect the rate of metamorphosis when added at the early stage, whereas middle and late larvae treated with 0.5 µg ml^−1^ DD encountered a significant delay in metamorphosis with respect to controls in which the majority of larvae had completely metamorphosed into juveniles. Indeed, one day after exposure, late larvae treated with DD did not completely metamorphose, as shown by an increase in the percentage of late larvae or larvae during tail regression to 72% with respect to 20% in controls and a concomitant decrease of juveniles to 22% with respect to 78% in controls (figure 1*a*). Late larvae (2–4 h post-hatching) were also treated with different concentrations of DD (0.125, 0.25, 0.5, 0.9, 1.35 µg ml^−1^ corresponding to 0.8, 1.6, 3.2, 5.9, 8.9 µM, respectively) and examined for the rate of metamorphosis (figure 1*b*). At lower concentrations, DD did not significantly affect the rate of metamorphosis, whereas at 0.5 µg ml^−1^, we observed a significant delay in metamorphosis at 24 h post-treatment, as assessed by the drastic reduction in the number of juveniles with respect to the control. After 3 days, 72 h post-treatment, larvae treated with 0.5 µg ml^−1^ DD were weakly attached to the substrate, had not completely retracted their tail, whereas head reorganization proceeded normally (figure 1*c*). At 0.9 µg ml^−1^, DD had completely blocked metamorphosis, with 97% of late larvae and only 3% of juveniles with respect to the control. At higher concentrations (1.35 µg ml^−1^), the effect was toxic for larvae, with DD hampering larval development and tail retraction (figure 1*d*).

### 4.2. Decadienal induces changes in nitric oxide levels and redox homeostasis

**Figure 2.**
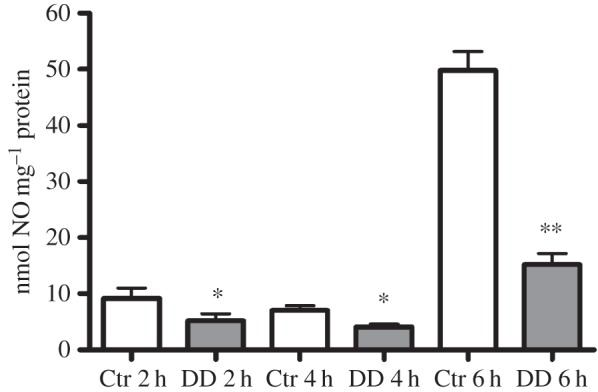
Decadienal (DD) induces a reduction in NO levels. Larvae treated with 0.5 µg ml^−1^ DD were harvested after 2, 4 and 6 h of treatment and processed for DAN assay. Results are representative of three independent experiments. Data are expressed as means ± s.e.m. and assessed by unpaired *t*-test. Asterisks represent significant differences with respect to the control: *p < 0.05, **p < 0.01.

**Figure 3.**
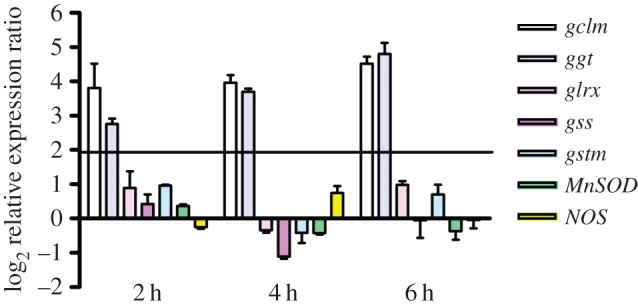
Gene regulation in response to decadienal (DD) treatment at different times of larval development. Histograms show the differences in expression levels of analysed genes, according to real-time qPCR. Middle larvae incubated with 0.5 μg ml^−1^ DD were collected after 2, 4 and 6 h. Data are reported as fold differences compared to the control (larvae in seawater without DD; mean ± s.d.). Fold differences equal to or greater than ±2 were considered significant.

**Figure 4.**
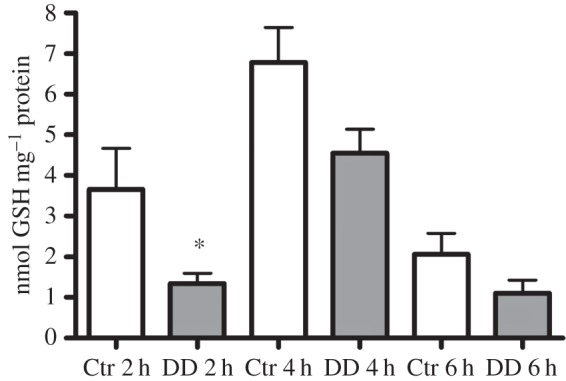
Decadienal (DD) induces a reduction in GSH levels. Larvae treated with 0.5 µg ml^−1^ DD were harvested after 2, 4 and 6 h of treatment and processed with the GSH assay kit. Results are representative of three independent experiments. Data are expressed as means ± s.e.m. and assessed by unpaired *t*-test. Asterisk represents the significance with respect to the control: *p < 0.05.

We have previously demonstrated that the endogenous modulation of NO levels in *Ciona* larvae affects the rate of metamorphosis [29]. In order to understand if NO mediates the response of the larvae to the toxin, we measured NO levels in *Ciona* larvae treated with the sublethal concentration of 0.5 µg ml^−1^ DD. NO levels significantly decreased after different times of DD treatment (figure 2). To understand if the change in NO levels was related to the fine regulation of redox homeostasis in response to DD, we first analysed the expression patterns of some genes involved in redox homeostasis. These genes were identified by *in silico* analysis of the *Ciona* genome and are reported in table 1. These genes include those coding for manganese superoxide dismutase (*Mn-SOD*), a key enzyme involved in the scavenging of reactive oxygen species [36,37], NO synthase (*NOS*)*,* responsible for NO synthesis [26,38], two enzymes responsible for GSH synthesis, the glutamate–cysteine ligase regulatory subunit (*gclm*) and GSH synthase (*gss*) [32,33], glutaredoxin-1 (*glrx*) involved in GSH reduction [32,33], gamma-glutamyl tranpeptidase (*ggt*) involved in GSH metabolism and recycling [39,40], and GSH *S*-transferase (*gstm*), which catalyses the conjugation of GSH to xenobiotic substrates for detoxification [32,33]. The relative expression of all these genes was followed by real-time qPCR experiments, after treating middle larvae (20–21 hpf) with 0.5 µg ml^−1^ DD (figure 3) and collecting them after 2, 4 and 6 h of treatment. The expression of *gclm* and *ggt* progressively increased at all hours tested, whereas the expression of the other genes, including *NOS*, was unaffected. These results prompted us to measure total GSH levels in larvae treated with DD. A significant reduction in total GSH levels was observed after 2 h treatment (figure 4), whereas thereafter, GSH levels were comparable to the controls.

### 4.3. Decadienal affects ERK signalling

**Figure 5.**
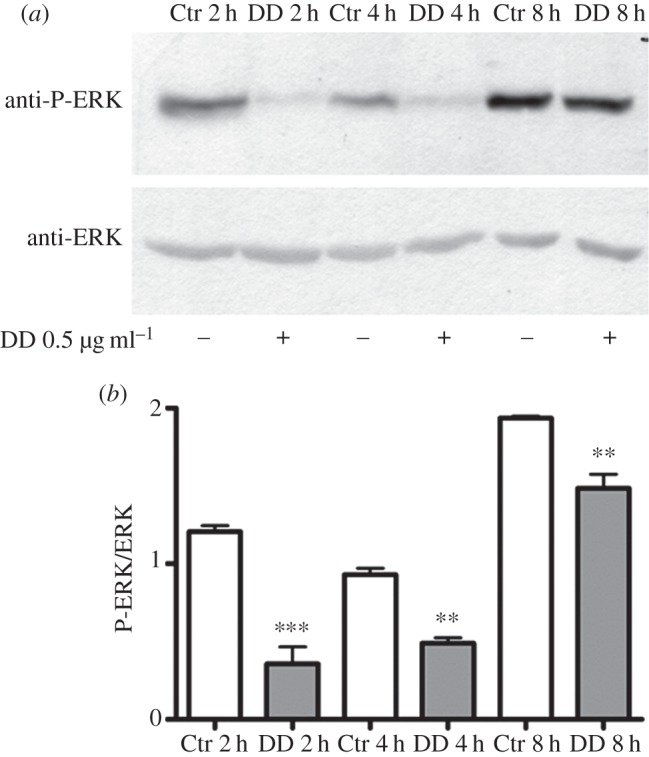
Decadienal (DD) induces ERK dephosphorylation. Middle stage larvae were treated with 0.5 µg ml^−1^ DD. Samples collected after 2, 4 and 8 h of treatment were examined for ERK activation. (*a*) Representative experiment showing a Western blot with anti-P-ERK and anti-ERK antibodies. (*b*) Histograms representing densitometric data analyses of P-ERK versus ERK intensity bands from three independent experiments reported as means ± s.e.m. and assessed by unpaired *t-*test. Asterisks represent significant differences with respect to the control: **p < 0.01, ***p < 0.001.

ERK activation represents a key event in *Ciona* life cycle transition. Its inhibition hampers the wave of apoptosis and subsequent metamorphosis of the ascidian [27,28]. We therefore decided to investigate the effect of DD on ERK phosphorylation. DD induced a significant decrease in P-ERK as early as 2 h post-treatment (figure 5*a*). The densitometric analysis of the P-ERK immunopositive bands with respect to the constitutive ERK band revealed a significant reduction of ERK phosphorylation at 2, 4 and 8 h after treatment (figure 5*b*).

To investigate if DD also affected gene transcription occurring downstream to ERK activation, we selected a series of ERK target genes mostly expressed in territories specialized for metamorphosis, such as palps and papillae, where *NOS* expression, NO production and ERK activation occur [24,25]. These genes include dehydrogenase with different specificities (*dhg*), ets-related transcription factor (*ets*) and matrix metalloprotease-24-precursor (*mx*). We also selected the specific map kinase phosphatases *mkp1* and *mkp3*, which we previously demonstrated to be regulated by NO during *Ciona* larval development [30]. Finally, we used *sushi*, a JNK target, the expression of which is necessary to induce apoptosis of tail cells at the onset of metamorphosis in *Ciona* [28], as an unrelated gene (table 1).

**Figure 6.**
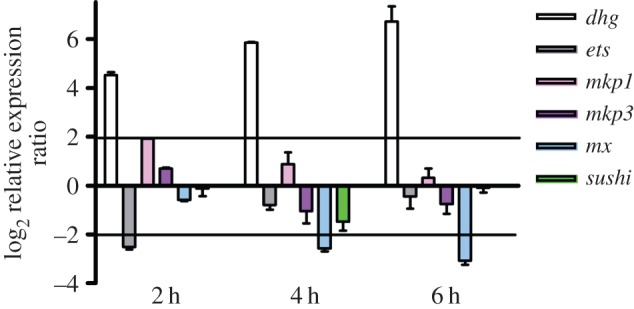
ERK-related gene regulation in response to decadienal (DD) treatment at different times of larval development. Histograms show the differences in expression levels of analysed genes, as revealed by real-time qPCR. Middle larvae incubated with 0.5 μg ml^−1^ DD were collected at 2, 4 and 6 h post-treatment. Data are reported as fold differences compared to the control (larvae in seawater without DD, mean ± s.d.). Fold differences equal to or greater than ±2 were considered significant.

The relative expression of these genes was followed by real-time qPCR experiments, after treating middle larvae with 0.5 µg ml^−1^ DD (figure 6). Larvae were collected at 2, 4 and 6 h of treatment. The expression of *dhg* started to progressively increase after 2 h and until 6 h of treatment, whereas *ets* and *mx* were downregulated after 2 h and 4–6 h treatment, respectively. *mkp1* was found to be upregulated after 2 h treatment, whereas the expression of the other genes, *mkp3* and *sushi*, was unaffected.

**Figure 7.**
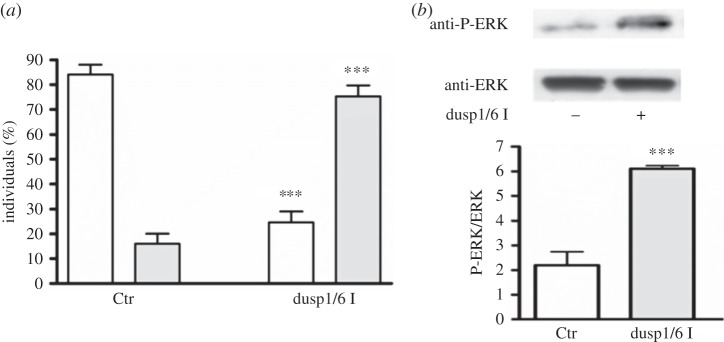
Mkp1 inhibition induces larval settlement. Hatched larvae were treated with 0.25 μM mkp inhibitor dusp1/6 I and examined after 4 h of treatment for larval settlement (*a*) and ERK activation (*b*). (*a*) The number of swimming larvae (white bars) and larvae just attached to the substrate (grey bars) were counted and reported as percentage of the total. (*b*) Western blot with anti-P-ERK and anti-ERK antibodies and relative histograms representing densitometric data analyses of P-ERK versus ERK intensity bands. Results are representative of five independent experiments. Data are expressed as means ± s.e.m. and were assessed by unpaired *t*-test. Asterisks represent significant differences with respect to the control: ***p < 0.001.

To confirm the role of mkp1 in the regulation of ERK activity during larval development, we treated hatched larvae with 0.25 µM of the dual-specific phosphatase inhibitor (dusp 1/6 I). After 4 h of treatment, the inhibitor caused an increase in larval settlement, as assessed by the significant increase of larvae attached to the substrate and the concomitant decrease of swimming larvae with respect to the control (figure 7*a*). The specificity of the inhibitor for ERK activity was confirmed by the increase of ERK phosphorylation (figure 7*b*).

## 5. Discussion

Metamorphosis of marine organisms represents an evolutionarily advantageous process that allows life cycle transition from the vegetative to reproductive stage when environmental conditions are favourable for offspring survival. Briefly, the vegetative stage consists of swimming larvae that acquire the competence to receive specific environmental cues to induce the settlement and the initiation of metamorphosis into the reproductive adult. The binding of environmental cues to cell surface receptors of larvae activates molecular pathways that finally lead to an extensive transformation of the body plan through apoptotic processes. NO plays a key role in these transformations, by regulating the timing of life cycle transitions in several marine organisms, including ascidians [26,29,30,41–48]. During *C. intestinalis* larval development, NO is known to affect different molecular targets. Briefly, NO affects caspase-3 activity [26], induces ERK and P-ERK nitration [29], and decreases MAPK phosphatase levels with consequent ERK activation [30].

In this paper, we report that *Ciona* larvae respond to the diatom-derived toxic aldehyde 2,4-*trans*-decadienal (DD) when they become competent to sense environmental stimuli and can choose a favourable substrate to attach to and initiate metamorphosis. Larvae may come into contact with DD after the sinking and sedimentation of diatom blooms when concentrations of this aldehyde can be high enough to affect the initial stages of metamorphosis [10]. This is of considerable ecological relevance considering the importance of diatom blooms in nutrient-rich aquatic environments.

This study provides first insights into the molecular mechanisms that mediate the toxic effects of DD on *Ciona* larval development and initial stages of metamorphosis, by highlighting the involvement of the NO/ERK pathway.

First of all, we report that DD induces a redox unbalance in *Ciona* larvae, owing to changes in GSH homeostasis. GSH is the most abundant antioxidant molecule in cells, playing a key role in cellular defence mechanisms, owing to its ability to reduce oxidized compounds [49]. Therefore, GSH contributes to maintain integrity of cell membranes and organelles, and a correct rate of metabolism and growth. The early reduction of total GSH levels after DD treatment can be explained considering that DD is a hydroxyalkenal compound that can reduce intracellular GSH content, as already reported in human erythroleukaemia and in human lung carcinoma [50,51]. DD can rapidly react with thiol compounds such as GSH to form conjugated complexes with consequent inhibition of some enzymatic activities and final induction of cytotoxicity. In our system, GSH depletion could be responsible for a transient loss of cell membrane integrity leading to calcium unbalance, with consequent NOS inhibition and reduction of endogenous NO levels. In particular, DD does not induce a transcriptional regulation of *Ciona NOS* gene, suggesting a post-translational regulation of *Ciona* neuronal NOS by a calcium/calmodulin signal [52]. The progressive upregulation of the two genes involved in GSH metabolism, *gclm* and *ggt*, suggests the activation of a detoxification mechanism in larvae against DD, which can counteract the deleterious effects of DD, thus contributing to the subsequent restoration of GSH levels.

Our finding that DD causes a dose- and time-dependent delay in metamorphosis with a concomitant decrease in ERK phosphorylation closely resembles what happens when endogenous NO levels are reduced by treating larvae with NOS inhibitors or NO scavengers [29,30]. Indeed, also in this case, DD causes a reduction in NO levels, leading to the upregulation of *mkp1*, the gene coding for the specific MAPK phosphatase responsible for ERK dephosphorylation in *Ciona* [30]. The fact that inhibition of this phosphatase induces ERK activation and larval settlement highlights the key role of ERK signalling in deciding the time of substrate attachment. The reduction in ERK phosphorylation upon DD exposure could represent a defence strategy to avoid the attachment of larva to unfavourable substrates such as those closest to toxic diatom blooms.

The DD-induced decrease in ERK phosphorylation also affected the expression of ERK target genes, including the metabolic enzyme dehydrogenase *dhg* and the developmental genes *ets* and *mx*. The progressive upregulation of *dhg* may be also due to the activation of a detoxification mechanism by larvae against this aldehyde, as already occurs with genes involved in GSH homeostasis. On the other hand, the downregulation of *ets* and *mx* could explain the weaker attachment of papillae to the substrate and the delay in tail retraction in most of the larvae treated with DD. Indeed, *ets* encodes a transcription factor that regulates the expression of genes involved in several developmental processes [53]. On the other hand, *Mx* encodes a metalloprotease involved in the composition and remodelling of the extracellular matrix during embryonic development [54,55]. Its regulation has been shown to affect apoptosis during amphibian metamorphosis [56]. Moreover, our finding that DD regulates *ets* expression levels via NO-mediated ERK activation is in agreement with a previous report in breast cancer [57].

**Figure 8.**
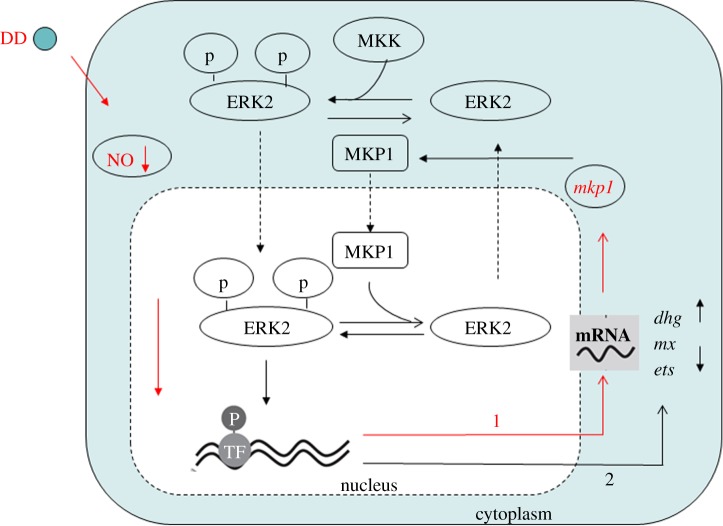
Proposed model of NO signalling induced by decadienal (DD). DD induces the reduction of endogenous NO levels that leads to the early upregulation of *mkp1* (line 1). Mkp1 is responsible for ERK dephosphorylation and downstream changes in gene transcription of the ERK target genes, *dhg*, *mx*, *ets* (line 2).

The above results can be schematically summarized in a model in which NO and ERK signalling cross-talk together in response to DD exposure (figure 8). DD induces the reduction of endogenous NO levels likely owing to GSH depletion. A reduction in NO levels induces the early upregulation of mkp1 with consequent ERK inactivation and changes in downstream transcription of the metabolic enzyme dehydrogenase *dhg* and the key developmental genes *ets* and *mx*, thereby causing a delay in metamorphosis.

## 6. Conclusion

*Ciona* is a broadcast spawning marine invertebrate which usually invests in a single annual spawning event in early spring [58] and should synchronize reproduction with specific environmental conditions to enhance the probability of survival. Diatoms also have an annual cycle, with blooms prevalent during the spring and early summer. Therefore, if *Ciona* gamete release coincides with a heavily grazed diatom bloom, PUAs may limit larval development and consequent metamorphosis, thereby affecting recruitment rates of future generations of this important benthic invertebrate. On the other hand, *Ciona* could time egg release prior to diatom blooms, so that larvae could successfully attach to the substrate and initiate metamorphosis, thereby avoiding contact with toxins [59]. However, because *Ciona* spawning period is very variable, depending on many environmental factors, such as temperature and light conditions, both hypotheses may be valid.

Our results show, for the first time, that ERK signalling is a key target of DD, thereby opening new perspectives on the study of the molecular pathways affected by PUAs. Our results also show that when larvae are continuously exposed to DD they weakly attach to the substrate and do not completely retract their tail, thus confirming that, during larval development, ERK activation is necessary, first in the papillae for the correct larval settlement and then in the tail for the initiation of the apoptotic wave preceding tail retraction. In this context, DD affects the decision and the time in which larvae choose the favourable substrate to attach to and go into metamorphosis. Future studies will be devoted to validating the results obtained with the pure molecule, exposing mature *Ciona* adults to natural decadienal-producing diatoms.
